# Two Lung Cancer Development-Related Genes, Forkhead Box M1 (*FOXM1*) and Apolipoprotein E (*APOE*), are overexpressed in Bronchial of Patients after Long-Term Exposure to Sulfur Mustard

**Published:** 2017

**Authors:** Eisa Tahmasbpour, Mostafa Ghanei, Yunes Panahi

**Keywords:** Apolipoprotein E, Cancer, Forkhead box M1, Lung, Oxidative stress, Sulfur mustard

## Abstract

Sulfur mustard (SM) is a strong alkylating and mutagenic compound that targets human airway system. We considered the expression of Forkhead box M1 (*FOXM1*) and apolipoprotein E (*APOE*) genes, which are responsible for cell proliferation, differentiation, tumorigenesis, and increased risk of lung cancer, in the lung bronchial tissue of patients exposed to SM. After performing pulmonary functional tests (PFTs), 11 human subjects (five controls and six SM-exposed patients) were entered in this study. Total RNA were extracted from all biopsy samples and then cDNA was synthesised for each specimen using RT-PCR. Changes in gene expression were measured using a RT^2^ Profiler ™PCR Array. PFTs have demonstrated more obstructive and restrictive spirometric patterns among patients compared to the controls. A higher expression was recorded for both examined genes in bronchial of SM-exposed patients. Expression of *FOXM1* and *APOE* genes in bronchial of the patients was significantly (*p* < 0.001) overexpressed by 14.8316 and 3.9504-folds, respectively. Mustard lungs were associated with increased expression of *FOXM1* and *APOE* genes, which suggests an increased risk of lung cancer among these patients. Since *FOXM1* and *APOE* are considered as oxidative stress responsive genes, we speculate that increased expression of these genes is more likely linked to overproduction of reactive oxygen species (ROS) and oxidative stress (OS) in mustard lungs. Further studies are required at protein level among SM-exposed patients with lung cancer to use these genes as lung cancer biomarkers among these patients.

## Introduction

Sulfur mustard (SM) or 2, 2›-Dichlorodiethyl sulfide, is an oily lipophilic substance which had been used as a chemical warfare agent. The highest unconventional application of SM occurred in Iran-Iraq war (1980-1988) that injured more than 100,000 Iranians (1, 2). Over 50,000 survivors of SM attacks remain alive in Iran and as a group suffer from high rates of chronic illnesses, particularly inflammatory conditions of the respiratory system, skin and eye (3-5). SM has several pathological effects in various from reproductive organs to respiratory systems, which have previously been reported (2, 6). The respiratory system is the major target of SM toxicity following inhalation exposure, which occurs in a dose-dependent manner from the nasal mucosa to the terminal bronchioles (7). These adverse effects are often lethal in the short term, and a source of ongoing symptoms and disability in long-term survivors. 

Nevertheless, the cellular and molecular mechanisms of SM toxicity on respiratory function still have not been fully recognized. One of these mechanisms is related to increased oxidative stress (OS) induced by reactive oxidative species (ROS). SM accelerates oxidative stress (OS) through either an increase reactive oxidative species (ROS) generation from endogenous or a decrease in antioxidant capabilities and oxidative DNA repair (5). This oxidative stress then, in turn, may damage DNA, resulting in chromosome instability, modify gene expression, genetic mutation or modulation of cell growth that may cause cell death and tissue damage (5). It may alter remodeling of extracellular matrix, apoptosis, and mitochondrial respiration, cell proliferation, maintenance of surfactant, and protease activity as well as effective alveolar repair responses and immunity modulation in lung tissue which is associated with inflammation and tissue damage (5). 

Altered expression of different genes, particularly oxidative stress responsive genes, is the major mechanism for direct effects of SM exposure on respiratory system. Overexpression or downregulation of these genes in OS conditions may alter cells proliferation and function. In this study, we considered altered expression of Forkhead box M1 (*FOXM1*) and apolipoprotein E (*APOE*) genes in mustard lungs. *FOXM1* is an OS responsive gene that stimulates cell proliferation and cell cycle progression by promoting the entry into S and M phases (8, 9). It is also involved in tumorigenesis, tumor initiation, growth, progression, and tumor-associated lung inflammation (10). *APOE* is also an OS responsive gene with antioxidative properties; however, recent studies have demonstrated that *APOE* overexpression promotes cancer proliferation and migration and contributes to an aggressive clinical course in patients with lung adenocarcinoma (11, 12). Since mustard lungs continuously generate high levels of ROS from both mitochondrial and extramitochondrial sources, it may increase the expression of the *FOXM1* and *APOE* genes in lung bronchial of injured patients. Therefore, overexpression of *FOXM1* and *APOE* due to increased level of ROS and OS may induce the risk of lung cancer in these patients. However, epidemiological studies revealed an association between SM exposure and the risk of developing respiratory tract cancer (13). To the best of our knowledge, there is no report of *FOXM1* and *APOE* genes expression in lung bronchial tissue of SM exposed patients. As part of ongoing research into the mechanism of action of SM, the present study was performed to examine whether the expression of *FOXM1* and *APOE* was altered in lung bronchial of patients who exposed to SM.

## Experimental

*Subjects*

Lung biopsy samples were obtained from SM-exposed patients and normal subjects undergoing bronchoscopy for various reasons. The patients were individuals who had a documented encounter with SM during the Iran-Iraq war. Normal subjects were individuals who undergone bronchoscopy for various reasons; however, they didn’t any pulmonary problem after consideration by pulmonologists. Our pulmonologists found them as healthy subjects after carefully considerations of biopsies by H&E staining and also bronchoscopy evaluation. The Ethics review board of the Baqiyatallah University of Medical Sciences approved the study and all subjects signed an informed consent form. Prior to sample collection, demographic and basic clinical characteristics of all subjects including weight, high, BMI, blood pressure, body temperature, and breathe rate were considered. Pulmonary function tests (PFT) including: FVC, FEV1 and FEV1/FVC were also determined by spirometry. In this study, we included patients with moderate lung injury (FEV1 > 50% and FEV1 < 80%; FEV1/FVC > 50% and FEV1/FVC < 80%). Disease severity was determined by combining the current level of symptoms, pulmonary function, and maintenance treatment. Subject who met the following criteria were excluded: other chronic lung diseases (*e.g.* asthma or allergy), bronchiectasis or pneumonia, cardiovascular disease, autoimmune disease (*e.g.* rheumatoid arthritis), lung cancer, diabetes mellitus, drug addictions, elderly people (older than 65 years old), smokers, organ transplant recipients and patients with history of occupational pulmonary exposure to other toxic agents. At the time of the examination, all patients were clinically stable and had not experienced any use of anti-inflammatories or antioxidant drugs (*e.g.* N-acetyl cysteine) for at least one month prior to study. Accordingly, six exposed male patients with moderate SM-lung injury and five healthy male subjects who full-filled the inclusion criteria were enrolled in the study. Mean time after SM exposure among our patients was 27.33 ± 0.57 years. The mean standard ages of patients and controls were 58.0 ± 10.81 and 48.33 ± 24.58 years, respectively. 

*Lung biopsy specimens*

The process of tacking lung biopsies is described previously in our works (14). Briefly, bronchoscopy was performed under O_2_ saturation at 5 L/min by nasal catheter. All subjects were premedicated intramuscularly with atropine (0.5 mg) and diazepam (10 mg) and orally with dihydrocodein (10 mg). Nares and oropharynx were topically anesthetized with 10% lidocaine before bronchoscopy. After local anesthesia, the flexible fiberoptic bronchoscope (PENTAX, EPK-100p, with a 2.0 mm diameter channel) was introduced and tissue samples were taken using bronchoscopic forceps (Olympus). All biopsy specimens were taken at the origin of the bronchial subcarinal segments. Vital signs, electrocardiograph (EKG) output and oximetry were recorded throughout the procedure. All bronchoscopy procedures were performed by the same experienced bronchoscopist surrounded by the same research team. Three biopsy samples were taken from each patient, and were immediately immersed in RNA Later reagent (R0901 SIGMA, USA). The samples in RNA Later reagent were stored at −80 °C until RNA extraction. 

*RNA extraction*

Total RNA was isolated from lung biopsy samples using the RNX-Plus (SinaClon; RN7713C) Kit and then further purified by RNeasy Mini Kit (Qiagen; Cat No-74104), according to the manufacturer’s instructions. The quantity and quality of extracted RNA were determined using the Nanodrop ND-1000 spectrophotometer (Thermo Sci., Newington, NH). Electrophoresis a fraction of each RNA sample on 1% denaturing agarose gel stained with ethidium bromide was used to analyze the quality of the RNA samples. The isolated RNA was stored at −80 °C for the further considerations.

**Figure 1:**
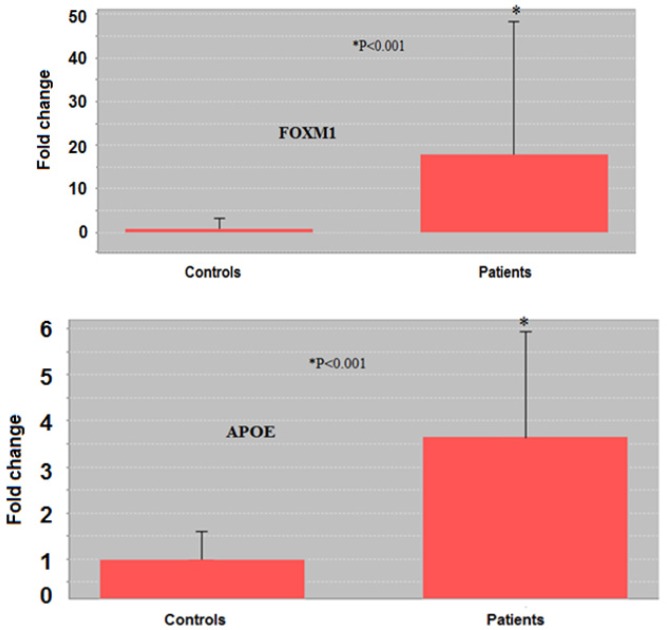
Comparison of *FOXM1* and *APOE* genes expression between control and SM-exposed groups by fold-change. The expression of *FOXM1* and *APOE* genes in the bronchial of patients was significantly (*p* < 0.001) higher than in controls. ^*^p < 0.05 is considered as significant difference

**Figure 2:**
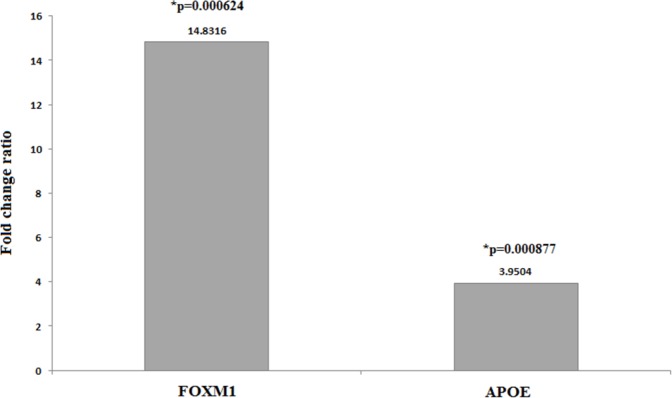
Fold changes ratio of the *FOXM1* and *APOE* expression in patients to the controls. SM-exposed patients showed expression of *FOXM1* 14.8316 and *APOE* 3.9504-folds higher (*p* = 0.000624 and *p* = 0.000877, respectively) than those of controls that reveal

**Figure 3:**
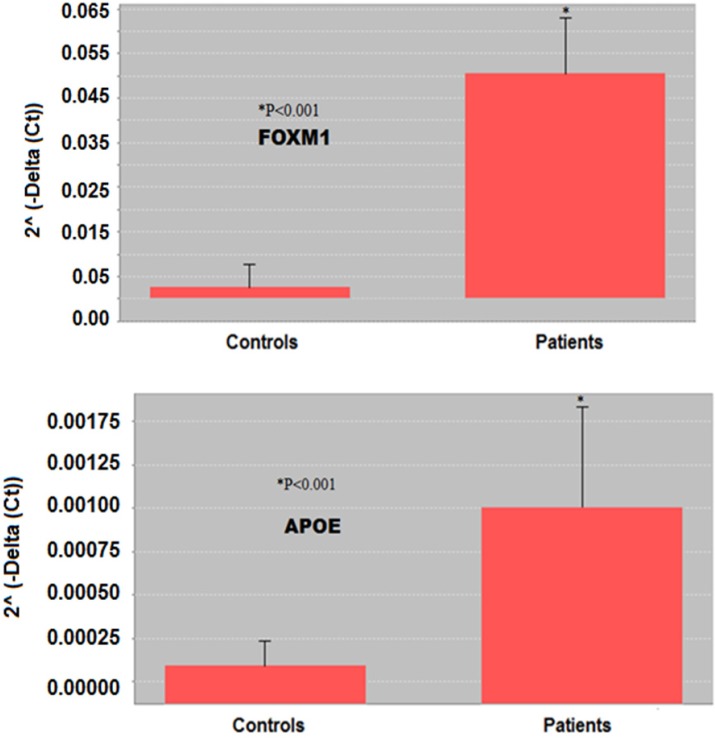
Comparison of *FOXM1* and *APOE *expression between controls and SM-exposed patients by 2^˄ ^(-Delta (Ct)). All RT-PCR experiments were performed in three independent experiments conducted in triplicate. Based on gene 2^˄ ^(-Delta (Ct)), the expression of *FOXM1* and *APOE* genes in lung tissue of the patients was significantly (*p *< 0.001) higher than in controls. ^*^p <0.05 is considered as significant difference

**Figure 4:**
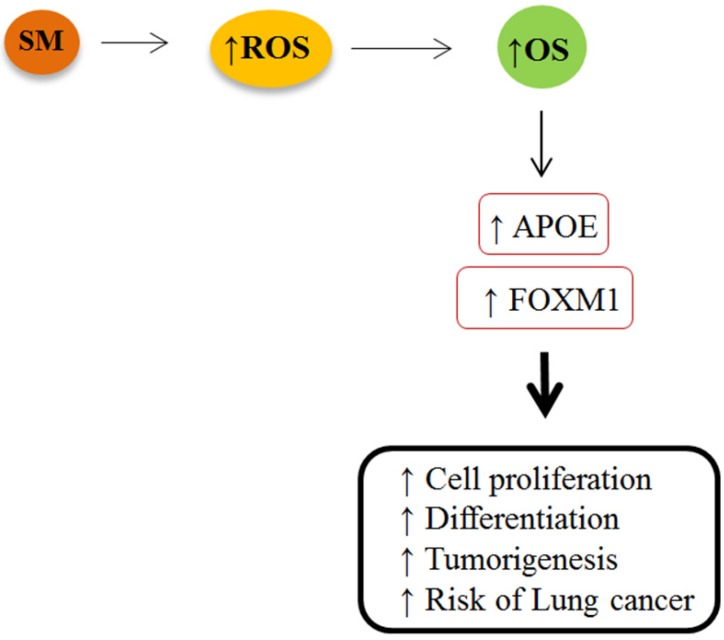
Increased expression of *FOXM1* and *APOE* genes is likely linked to overproduction of ROS and OS in mustard lungs that may increase risk of lung cancer among these patients. SM: sulfur mustard; ROS: reactive oxygen species; OS: oxidative stress; *APOE*: apolipoprotein E; *FOXM1*: Forkhead box M1

*Measuring gene expression using the PCR Array*

Changes in expression of *FOXM1* and *APOE* genes in the bronchial of all samples were measured using RT^2^Profiler™ PCR Array (Qiagen; PAHS-065ZA-6), as it described in our previous study (14). It consisted of the following steps: a) Clean-up of the isolated RNA in order to remove contaminating DNA followed by cDNA synthesis using RT^2^ First Strand Kit, b) Confirmation of the presence of cDNA in the samples using human RT^2^ RNA QC PCR Array, c) Quantitative analysis of gene expression in the samples using RT^2^ Profiler™ PCR Array, including an ABI7500 cycler and RT^2^ SYBR Green/ROX qPCR Master Mix, d) Data analysis using the ΔΔCt method, including RT^2^ Profiler PCR Array data analysis software version 3.5 (http://pcrdataanalysis.sabiosciences.com/pcr/arrayanalysis.php).

After RNA extraction, cDNA was synthesised from the equal amounts of RNA (1 μg) using the RT^2^ First Strand kit (Qiagen; Cat No-330401). An equal amount of cDNA was mixed with RT^2^ SYBR Green/ROX qPCR Mastermix (QIAGEN Company, Cat No: 330522), and distributed to each PCR array well containing portions of specific genes. PCR of study genes was performed in 96-well plates, according to manufacturer’s instructions. This array contained 12 control genes, including five housekeeper genes for normalization: Actin beta (ACTB), Beta-2-microglobulin (B2M), Glyceraldehyde-3-phosphate dehydrogenase (GADPH), Hypoxanthine phosphoribosyl transferase 1 (HPRT1), Ribosomal protein, large, P0 (RPLP0), as well as one negative control to monitor the human genomic DNA contamination (HGDC). Three reverse transcription control (RTC) wells were used to check for RT reaction efficiency with qPCR test. The PCR array also contained three wells of positive PCR controls (PPCs) to determine the efficiency of the polymerase chain reaction. These controls use an artificial DNA sequence predefined in the detection process. Replicated control wells (RTC and PPC) also assess the consistency among wells and plates.

*Statistical analysis*

An independent *t*-test was considered to compare each of the parametric data between two groups using SPSS software (IBM, version 19). A probability of less than 0.05 was considered as significant. Biological relevance of differently expressed genes was investigated using RT^2^ Profiler PCR Array Data Analysis software, version 3.5 (http://pcrdataanalysis.sabiosciences.com/pcr/arrayanalysis.php). The Wilcoxon–Mann–Whitney test was applied to validate the homogeneity of the reaction of expression of each gene (*p *< 0.05). For comparison between the two groups, the software calculated the quantification cycle threshold (Ct) of the patient group (IRG) in relation to the Ct of the control group (CG) expressed in the logarithm basis. The gene expression results are shown as positive/upregulation expression (IRG higher than CG) or negative/downregulation expression (IRG lower than CG). 

## Results

*Spirometry tests*

There was no overall difference in baseline or demographic characteristics such as age, BMI and blood pressures between two groups. PFTs data have demonstrated more obstructive and restrictive spirometric patterns among patients compared to the control group. The mean value of FEV1in patients and control groups were 71.66 ± 4.04% and 95.33 ± 12.09%, respectively (*p *= 0.032). Control groups (95.72 ± 9.5%) demonstrated significantly higher FVC value (*p *= 0.037) than any patients groups (77.79 ± 3.38%). The mean ratio of FEV1 to FVC among patients and controls was 72.66 ± 8.5% and 104.42 ± 11.78%, respectively (*p* = 0.019). 

*Gene expression study*

Figure 1 shows a comparison of *FOXM1* and *APOE* genes expression between control and SM-exposed groups by fold-change. A higher expression was recorded for *FOXM1* and *APOE* genes in the bronchial of SM-exposed patients (*p* < 0.001).

Figure 2 depicts fold changes ratio of the examined genes expression in patients to the controls. All lung biopsy samples of SM-injured patients were found to overexpress *FOXM1* and *APOE* genes at the highest level. SM-exposed individuals demonstrated expression of *FOXM1* 14.8316 (*p* = 0.000624) and *APOE* 3.9504-folds (*p* = 0.000877) higher than those of controls that reveal.

Comparison of studied genes expression between control and SM-exposed groups based on 2˄ (-Delta (Ct)) is also demonstrated in Figure 3. Based on gene 2˄ (-Delta (Ct)), expression of both *FOXM1* and *APOE* genes in bronchial of the patients was significantly (*p* < 0.001) higher than in controls.

## Discussion

The results of the current study demonstrated an altered expression of *FOXM1* and *APOE* genes in the bronchial of SM-injured patients. We discovered a significant rise in expression of *FOXM1* and *APOE* genes in the bronchial of the patients, suggesting an increased ROS production and oxidative stress mustard lungs. Forkhead box M1 is known as one of the most upregulated oxidative stress responsive genes, which is expressed in actively dividing cells. It is expressed in a variety of respiratory cell types undergoing DNA replication and mitosis (9). The *FOXM1 *regulates expression of genes that are involved in cell proliferation, differentiation, and transformation by promoting both G1/S and G2/M transition (15). Previous studies demonstrated that overexpression of *FOXM1* is associated with tumorigenesis, apoptosis and progression in a variety of human cancers, specifically lung cancer (16). More recent studies have considered the *FOXM1* as a novel biomarker of lung cancer because not only it stimulates proliferation and growth of cells, but also it is associated with high DNA replication rates of lung tumor cells (12). Increased expression of *FOXM1* in response to high level of ROS may be one reason for lung cancer in SM-victims. Numerous studies have revealed an association between respiratory exposure to SM and the increased risk of developing lung cancer (13, 17-19). Since SM-injured lungs accumulate high level of free radicals, elevated levels of ROS render injured lung cells more vulnerable to oxidative stress than normal cells. Although *FoxM1* protects damaged lung cells against adverse effects of oxidative stress by upregulating expression of scavenger enzymes, in higher expression it is associated with abnormal regulation mechanism of cell proliferation, accelerated apoptosis or cell death (Figure 4) (8). This is supported by our finding that SM-injured lung cells expressed greater constitutive levels of *FOXM1* compared with normal lungs. We therefore hypothesized that there is a tight link between overexpression of *FOXM1* and the possibility of increased risk of lung cancer among SM-injured patients. 

Mustard lungs also displayed increased expression of apolipoprotein E in mustard lungs. Similarly, overexpression of APOA1 was reported in bronchoalveolar lavage (BAL) fluid of SM exposed patients in a previous study (20). Since it exhibits antioxidant properties in lungs, we suggest that *APOE* overexpression protects mustard lungs from ROS-induced damages, by mitigating intracellular oxidation and exerting extracellular antioxidant properties. Nevertheless, recent data have implicated that long-term overexpression of *APOE* can promote cell proliferation and increased risk of lung cancer among patients (11).

We speculate that increased expression of *FOXM1* and *APOE* genes is more likely linked to overproduction of ROS and OS in mustard lungs that may increase the risk of lung cancer among these patients (Figure 4). Therefore, antioxidant therapy is recommended as a possible treatment to minimize the SM-induced oxidative stress among injured victims. However, further studies are necessary to consider the possible effects of antioxidants on expression of these genes. It is also important to note that the results being analyzed are gene expression data and does not reflect protein expression. Overexpressed genes don’t necessarily translate into higher protein expression levels. Epigenetics changes including creation of mutations in DNA sequence, phosphorylation or other post-translational alterations of proteins upon SM exposure may lead to structural and functional changes in protein. In addition, alterations in some miRNAs responsible for regulating post-translation events may inhibit the expression of the antioxidant proteins in the poisoned cells at translational level. Therefore, further studies at proteomics level are required to consider *FOXM1* and *APOE* proteins in mustard lungs. 
